# Biomarker-Enriched Host-Directed Trials in Sepsis: Integrating Immune, Endothelial, Coagulation, Redox, and Metabolic Domains

**DOI:** 10.3390/cimb48101068

**Published:** 2026-10-15

**Authors:** Juan S. Izquierdo-Condoy, Jhan S. Saavedra-Torres, Betty Liliana Coral Gaón, Alice Gaibor-Pazmiño, Janeth C. Gil, Daniel Armando Tenorio-Castro, Angelica Grey Sayas, Andrea Pérez Giraldo, Susana García-Cañarte, Marlon Rojas-Cadena, Valeria Estefania Galecio Tito

**Affiliations:** 1 One Health Research Group, Universidad de Las Americas, Quito 170124, Ecuador; 2 Facultad de Ciencias de la Salud, Fundación Universitaria Vision de las Americas, Pereira 660003, Colombia; 3 Facultad de Ciencias de la Salud, Universidad Tecnológica de Pereira, Pereira 660003, Colombia; 4 Facultad de Salud, Universidad Santiago de Cali, Cali 760035, Colombia; 5 Facultad de Ciencias de la Salud, Universidad del Quindio, Armenia 630004, Colombia; 6 Facultad de Ciencias de la Salud, Corporacion Universitaria Empresarial Alexander Von Humboldt, Armenia 630003, Colombia; 7 Clínica Medica, Hospital Fundación Sanatorio Güemes, Buenos Aires C1188, Argentina; 8 Departamento de Medicina Interna, Hospital de Especialdiades Eugenio Espejo, Quito 170403, Ecuador

**Keywords:** sepsis, endothelial dysfunction, immunometabolism, immunothrombosis, biomarkers, mitochondrial dysfunction, precision medicine

## Abstract

Sepsis is a life-threatening, biologically heterogeneous syndrome in which immune dysregulation, endothelial injury, coagulation activation, redox imbalance, and metabolic reprogramming contribute to microvascular and organ dysfunction. This structured narrative review integrates these five domains to inform biomarker-enriched trials of host-directed interventions, emphasizing the endothelium as an interface between inflammation, immunothrombosis, and impaired cellular bioenergetics. Candidate biomarkers of immune competence, endothelial injury, coagulation, and metabolism are examined alongside investigational immunomodulatory and restorative strategies. The proposed framework distinguishes three functional tiers: screening and biological enrichment; pharmacodynamic target engagement and safety assessment; and patient-centered outcomes. Candidate host-response profiles are hypothesis-generating, may overlap, and require prospective validation, with explicit provisions for mixed and indeterminate classifications. A complementary implementation model separates routine assessments from trial-specific assays and advanced mechanistic substudies, addressing sampling, reporting, missing measurements, and assay harmonization. Evidence remains heterogeneous and largely preclinical, observational, or early-phase; predictive thresholds, therapeutic windows, and patient-centered benefits remain unestablished for most candidate strategies. This framework supports prospective trial design rather than routine bedside treatment selection. Biomarker improvement must be evaluated alongside pathogen control, organ recovery, adverse events, and longer-term outcomes and should not be assumed to indicate clinical benefit.

## 1. Introduction

Sepsis is a life-threatening syndrome characterized by substantial biological heterogeneity, in which a dysregulated host response to infection drives organ dysfunction. Earlier models focused primarily on uncontrolled systemic inflammation, whereas current evidence supports a broader disruption involving inflammatory signaling, redox regulation, cellular metabolism, coagulation, and endothelial homeostasis [1,2]. Pathogen-associated and damage-associated molecular patterns activate pattern-recognition receptors, including Toll-like receptor 4, triggering NF-κB-dependent programs and the release of mediators such as tumor necrosis factor, interleukin-1β, and interleukin-6 [3,4]. These responses are essential for pathogen recognition and clearance, but when they become excessive, prolonged, or inadequately compartmentalized, they contribute to progressive tissue injury.

Under physiological conditions, immune activation is closely coupled with redox signaling and metabolic adaptation. Reactive oxygen species have a dual role: excessive production can damage cellular structures, whereas controlled generation functions as a signaling mechanism that influences transcription, antimicrobial activity, and cellular differentiation. Mitochondria support ATP production and participate in redox-sensitive innate immune signaling, including reactive oxygen species-dependent antimicrobial and inflammatory responses [5]. Immune cells can shift between oxidative phosphorylation and glycolysis according to their activation state and functional demands. This metabolic flexibility supports rapid antimicrobial responses while preserving cellular viability and tissue homeostasis [5,6].

In sepsis, these coordinated inflammatory, redox, and metabolic responses become disrupted. Mitochondrial injury, increased NADPH oxidase activity, reduced antioxidant capacity, and impaired redox control promote excessive or mislocalized reactive oxygen species production [7,8]. Inflammatory signaling further alters mitochondrial respiration and substrate use, producing cell- and phase-specific shifts toward glycolysis, impaired oxidative phosphorylation, and reduced metabolic flexibility [9,10,11,12,13,14]. When sustained, these changes compromise cellular energy production and resilience. Elevated lactate should not be attributed solely to tissue hypoxia or mitochondrial failure, as it may also reflect adrenergic glycolysis, altered substrate metabolism, or impaired hepatic and renal clearance. Persistent hyperlactatemia may nevertheless indicate ongoing physiological stress and is associated with worse outcomes [15,16].

The endothelium links these disturbances to organ dysfunction by regulating permeability, vascular tone, leukocyte trafficking, coagulation, and microvascular flow. In sepsis, glycocalyx shedding and junctional disruption promote capillary leak [17,18]. Complement activation, NETs, and platelet activation contribute to endothelial injury and microvascular thrombosis [19,20,21,22]. In this review, immunothrombosis denotes the reciprocal activation of innate immunity and coagulation within microvessels; the term does not imply that all coagulation abnormalities in sepsis have the same mechanism [20].

Sepsis does not follow a uniform progression from hyperinflammation to immunoparalysis. Proinflammatory and immunosuppressive responses may coexist from the early stages and evolve differently across tissues and cell populations. Thus, persistent cytokine signaling, endothelial activation, complement-mediated injury, and immunothrombosis can occur alongside lymphocyte depletion, impaired antigen presentation, reduced ex vivo cytokine responsiveness, and increased expression of inhibitory immune checkpoints [9,10,23,24,25]. Reduced monocyte HLA-DR expression may help identify an immunosuppressed phenotype, but no single biomarker captures the full immune status of an individual patient. This biological heterogeneity may partly explain why therapies targeting isolated mediators have generally failed to improve survival in unselected sepsis populations [26,27].

Host factors also influence the course of sepsis. Age, immunosenescence, diabetes, chronic kidney disease, malignancy, immunosuppressive treatment, and other chronic conditions may alter baseline inflammation, endothelial function, immune competence, and metabolic reserve [9,28,29]. These factors can affect pathogen clearance, vascular vulnerability, and the ability to restore homeostasis after the initial insult [30,31,32]. Infection source and severity, microbial burden, timing of antimicrobial therapy, and degree of organ dysfunction introduce additional variability. Sepsis is therefore better understood as a dynamic host-response syndrome than as a single biological state defined solely by pathogen burden or circulating cytokine levels.

These observations have important therapeutic implications. Broad suppression of inflammation may weaken antimicrobial defenses, whereas blockade of a single cytokine may be insufficient because of pathway redundancy, concurrent immunosuppression, and persistent endothelial or metabolic injury [26,27]. A potential translational approach is to combine prompt antimicrobial therapy and source control with serial assessment of immune, endothelial, and metabolic abnormalities. Biomarkers spanning these domains—including monocyte HLA-DR, ex vivo lipopolysaccharide responsiveness, cytokine trajectories, syndecan-1, soluble thrombomodulin, lactate kinetics, and cellular respiration indices—may eventually support this strategy. However, most still lack standardized assays, validated thresholds, rapid availability, and prospective evidence supporting treatment selection.

Precision trials should distinguish prognostic biomarkers, which identify patients at increased risk of adverse outcomes, from predictive biomarkers, which identify a differential response to a specific intervention. In sepsis, proteomic profiles have been associated with organ dysfunction and mortality, whereas transcriptomic subtypes have been proposed to identify subgroups that may respond differently to treatment; however, their value for treatment selection still requires prospective validation [33,34].

This review synthesizes evidence on immune, endothelial, coagulation, redox, and metabolic abnormalities in sepsis; evaluates candidate biomarkers for serial assessment; and examines the mechanisms, available evidence, and safety of host-directed interventions. Particular attention is given to endothelial injury because it links inflammatory signaling, coagulation activation, microvascular thrombosis, impaired cellular energy metabolism, and organ dysfunction. The proposed approach is intended to inform clinical-trial design rather than routine bedside treatment: prospectively defined biological abnormalities should be matched to interventions with relevant mechanisms, confirmed by measures of target engagement, and evaluated using patient-centered outcomes. The distinctive contribution of this review is its integration of five interdependent host-response domains—immune, endothelial, coagulation, redox, and metabolic—within a single translational framework focused on biomarker-enriched trial design. Rather than treating these pathways or candidate therapies in isolation, we link multidomain phenotyping to trial-specific biological enrichment, mechanism-specific target engagement, safety monitoring, and patient-centered outcomes. We further distinguish immediately feasible assessments from advanced mechanistic tools and emphasize serial reassessment as host-response phenotypes evolve over time.

## 2. Search Strategy and Methodological Approach

A structured narrative literature search was conducted in PubMed/MEDLINE, Scopus, and Web of Science from database inception to 1 June 2026. The search strategy combined controlled vocabulary and free-text terms related to “sepsis”, “septic shock”, “endothelial dysfunction”, “immunometabolism”, “redox signaling”, “oxidative stress”, “mitochondrial dysfunction”, “immunothrombosis”, “glycocalyx”, “neutrophil extracellular traps”, “complement”, “platelets”, “immune paralysis”, “biomarkers”, and “immunomodulatory therapy”. Additional searches were performed for specific therapeutic or regulatory pathways, including NLRP3, AMPK, SIRT1, SIRT3, Nrf2, HIF-1α, vagal neuroimmunomodulation, α7 nicotinic acetylcholine receptor signaling, specialized pro-resolving mediators, IL-7, GM-CSF, and immune-checkpoint modulation.

Human clinical studies, translational investigations, animal models, and mechanistic in vitro studies were considered when they directly addressed the predefined domains of the review. Priority was given to randomized or prospective clinical studies, human observational studies, systematic reviews, and recent mechanistic reviews. Seminal experimental studies were retained when required to explain foundational biological pathways or when human evidence was unavailable. Evidence from conditions other than sepsis was considered only when it provided relevant mechanistic support and was interpreted as indirect evidence rather than as proof of clinical applicability in sepsis.

Articles were selected according to their relevance to four predefined domains: (1) integration of inflammatory, redox, metabolic, and endothelial responses; (2) mechanisms of endothelial and immunothrombotic injury; (3) biomarkers with potential value for dynamic host-response phenotyping; and (4) restorative or immunomodulatory therapeutic strategies. Studies focusing exclusively on antimicrobial selection, infection-source management, or general supportive care were not reviewed in detail unless they informed the timing or safety of host-directed interventions.

The literature search and study selection were performed manually by two authors (J.S.S.-T. and A.G.-P.). Both authors independently screened retrieved records for relevance to the predefined domains of the review and assessed potentially relevant full-text articles. Disagreements regarding study relevance or inclusion were resolved through discussion and consensus. No automated screening algorithm, machine-learning classifier, artificial intelligence tool, or computational pipeline was used for literature screening or study selection. Reference lists of eligible articles and relevant reviews were additionally screened manually to identify potentially relevant studies not captured by the database searches.

Because this study was designed as a narrative translational review rather than a systematic or scoping review, no formal risk-of-bias assessment or certainty-of-evidence grading was performed. Nevertheless, the search was structured using predefined databases, search terms, review domains, and study-selection criteria to improve transparency and reproducibility.

The translational maturity of candidate interventions was described according to the highest level of evidence available, including preclinical, translational human, observational clinical, or early-phase interventional evidence.

## 3. Conceptual Framework: Integrated Immune, Endothelial, Coagulation, Redox, and Metabolic Dysregulation in Sepsis

### 3.1. Physiological Coordination of Inflammatory, Redox, and Metabolic Signaling

Physiological host defense depends on coordinated inflammatory, redox, and metabolic signaling. Reactive oxygen species function as controlled signaling intermediates that influence cytokine production, cellular activation, differentiation, and antimicrobial defense through reversible redox modifications of transcription factors and signaling proteins, including NF-κB and Nrf2 [5,7]. Their effects vary with concentration, cellular source, intracellular location, and duration of exposure. Accordingly, spatially and temporally restricted ROS production can support pathogen recognition, phagocyte activation, and microbial killing without causing widespread cellular damage.

Mitochondria contribute to this coordination by coupling ATP production with substrate metabolism, calcium handling, redox signaling, and antioxidant defenses [8,35]. Immune-cell metabolism adapts to functional demand: increased glycolytic flux supports rapid effector responses, whereas oxidative phosphorylation, fatty-acid oxidation, and mitochondrial biogenesis contribute to cellular persistence, resolution, memory, and repair. These programs are not mutually exclusive. Effective immune responses depend on the capacity to adjust their relative contribution according to the nature and duration of the stimulus.

Vascular homeostasis is also integral to this coordination. Endothelial cells regulate vascular tone, permeability, leukocyte recruitment, coagulation, and oxygen exchange, while nitric oxide signaling, glycocalyx integrity, mitochondrial function, and intercellular junctions preserve microvascular stability. Host defense therefore requires both an appropriate immune response and the maintenance of vascular integrity and sustainable cellular metabolism [9,10].

### 3.2. Immune, Redox, and Metabolic Dysfunction in Sepsis

During sepsis, inflammatory, redox, and metabolic pathways become quantitatively amplified and qualitatively disorganized. Excessive or prolonged ROS generation may result from mitochondrial dysfunction, NADPH oxidase activation, impaired antioxidant capacity, and altered nitric oxide metabolism. When redox buffering is exceeded, proteins, lipids, nucleic acids, and endothelial structures undergo irreversible oxidative modification, contributing to impaired signaling, barrier dysfunction, and reduced nitric oxide bioavailability [36,37].

Inflammatory mediators such as TNF, IL-1β, and IL-6 further promote endothelial activation, vascular permeability, and leukocyte recruitment, while mitochondrial injury may produce membrane-potential loss, altered electron transport, impaired ATP generation, and release of mitochondrial damage-associated molecular patterns [1,2,38]. These abnormalities may reinforce one another: inflammatory signaling disrupts mitochondrial function, mitochondrial products amplify innate immune activation, and oxidative injury further compromises endothelial and immune-cell performance.

The consequences are not limited to energy depletion. Mitochondrial dysfunction may alter apoptosis, calcium handling, inflammasome activation, epigenetic regulation, and immune-cell differentiation. Reduced metabolic flexibility may impair phagocytosis, antigen presentation, lymphocyte proliferation, and tissue repair even when systemic hemodynamics have been partially restored [10,39,40]. At the organ level, this disintegration contributes to vasoplegia, capillary leak, heterogeneous microvascular flow, impaired oxygen extraction, and progressive organ dysfunction.

In this review, loss of coordination refers to discordant trajectories among measurable biological domains rather than to a new clinical syndrome. Examples include persistent endothelial-injury markers despite declining circulating cytokines; reduced monocyte HLA-DR expression or ex vivo cytokine production in the presence of ongoing inflammatory activity; or persistent abnormalities in lactate kinetics or cellular respiration after correction of major macrocirculatory deficits. Such discordance can be evaluated only through prespecified serial measurements obtained at comparable time points and interpreted in relation to infection control, organ support, and renal or hepatic clearance. These patterns are proposed for trial stratification and require prospective validation; they should not be used as routine diagnostic categories or treatment targets.

### 3.3. Host-Response Heterogeneity and Etiologic Modifiers

The septic response is shaped by the causative organism, infection source, microbial burden, virulence, treatment timing, and baseline host characteristics. Different microbial ligands may preferentially activate distinct pattern-recognition receptors and generate partially different inflammatory, metabolic, and endothelial responses. Nevertheless, considerable biological overlap exists among Gram-positive, Gram-negative, fungal, and viral sepsis, preventing reliable assignment of fixed host-response phenotypes based on pathogen class alone.

Microbial products interact with infection severity, host comorbidities, immune history, and treatment timing to shape the evolving phenotype. Likewise, organ-specific injury reflects the combined effects of vascular susceptibility, inflammatory signaling, perfusion, infection source, and pre-existing organ reserve rather than pathogen tropism alone [3,13,41,42,43,44].

Aging, diabetes, chronic kidney disease, malignancy, immunosuppressive treatment, and other chronic disorders may modify the baseline endothelial and immunometabolic state. These conditions can reduce mitochondrial reserves, alter cytokine signaling, impair barrier repair, and promote metabolic inflexibility [9,28,29,30,31,32]. Consequently, similar infectious insults may produce different biological trajectories among patients and within the same patient as the disease evolves.

### 3.4. Coexisting Inflammatory and Immunosuppressive Programs

A central feature of sepsis is the coexistence of immune activation and functional immune failure. Persistent inflammatory signaling can occur simultaneously with lymphocyte apoptosis, impaired antigen presentation, reduced cytokine responsiveness, and increased expression of inhibitory receptors [13,45]. This pattern is better understood as the simultaneous evolution of distinct immune programs than as a fixed transition from early hyperinflammation to late immunoparalysis.

Initially protective regulatory mechanisms may become maladaptive when they are prolonged or excessive. IL-10, TGF-β, regulatory T-cell responses, tolerogenic or repair-associated macrophage programs, and immune-checkpoint pathways can limit collateral tissue injury during acute inflammation. Sustained activation, however, may suppress antigen presentation, T-cell proliferation, monocyte responsiveness, and antimicrobial effector function [24,46,47]. Upregulation of PD-1/PD-L1 and related inhibitory pathways may further reinforce functional exhaustion and reduce adaptive immune competence [48,49].

Immunoparalysis should therefore describe a clinically and biologically supported immunosuppressed phenotype, not all cases of late sepsis. Candidate features include persistent lymphopenia, reduced monocyte HLA-DR expression, diminished ex vivo cytokine production, impaired antigen presentation, checkpoint upregulation, viral reactivation, and recurrent secondary infection [48,49,50]. No single measurement is sufficient, and interpretation requires serial and multimodal assessment.

The coexistence of inflammatory and suppressive pathways may also account for the inconsistent effects of isolated cytokine blockade. In an unselected population, inhibiting one mediator may suppress a pathway required for pathogen control in some patients while having little biological relevance in others. Such treatment also leaves endothelial, coagulation, mitochondrial, and compensatory immune pathways largely unaffected [23,24,25].

### 3.5. Coordinated Regulation of Inflammatory and Metabolic Pathways

Pathogen-induced inflammation remains indispensable for microbial clearance. Phagocytosis, oxidative killing, complement activation, cytokine signaling, and antigen presentation are essential components of host defense [11,13,51]. The therapeutic objective should therefore not be the complete elimination of inflammation, but rather the restoration of proportionality, compartmentalization, and temporal control.

Antimicrobial treatment and source control remain fundamental because host-directed interventions cannot compensate for uncontrolled infection [21,52]. Conversely, pathogen elimination alone may not immediately reverse endothelial injury, mitochondrial dysfunction, immunothrombosis, or immune exhaustion. This supports an integrated model in which established sepsis care is combined, in research settings, with serial assessment of the host-response state.

Several pathways regulate specific inflammatory, redox, metabolic, and endothelial processes relevant to sepsis. NLRP3 inflammasome activation links danger sensing, caspase-1 activity, IL-1β and IL-18 maturation, pyroptosis, and endothelial injury [17,53,54]. AMPK, SIRT1, SIRT3, and Nrf2 contribute to mitophagy, mitochondrial turnover, mitochondrial biogenesis, antioxidant responses, metabolic adaptation, and endothelial resilience [7,55]. HIF-1α participates in metabolic and inflammatory reprogramming, although its effects vary across cell types, tissues, and disease phases. These pathways should not be interpreted as universally protective or harmful; their consequences depend on timing, magnitude, cellular compartment, and infection context.

**Figure 1:**
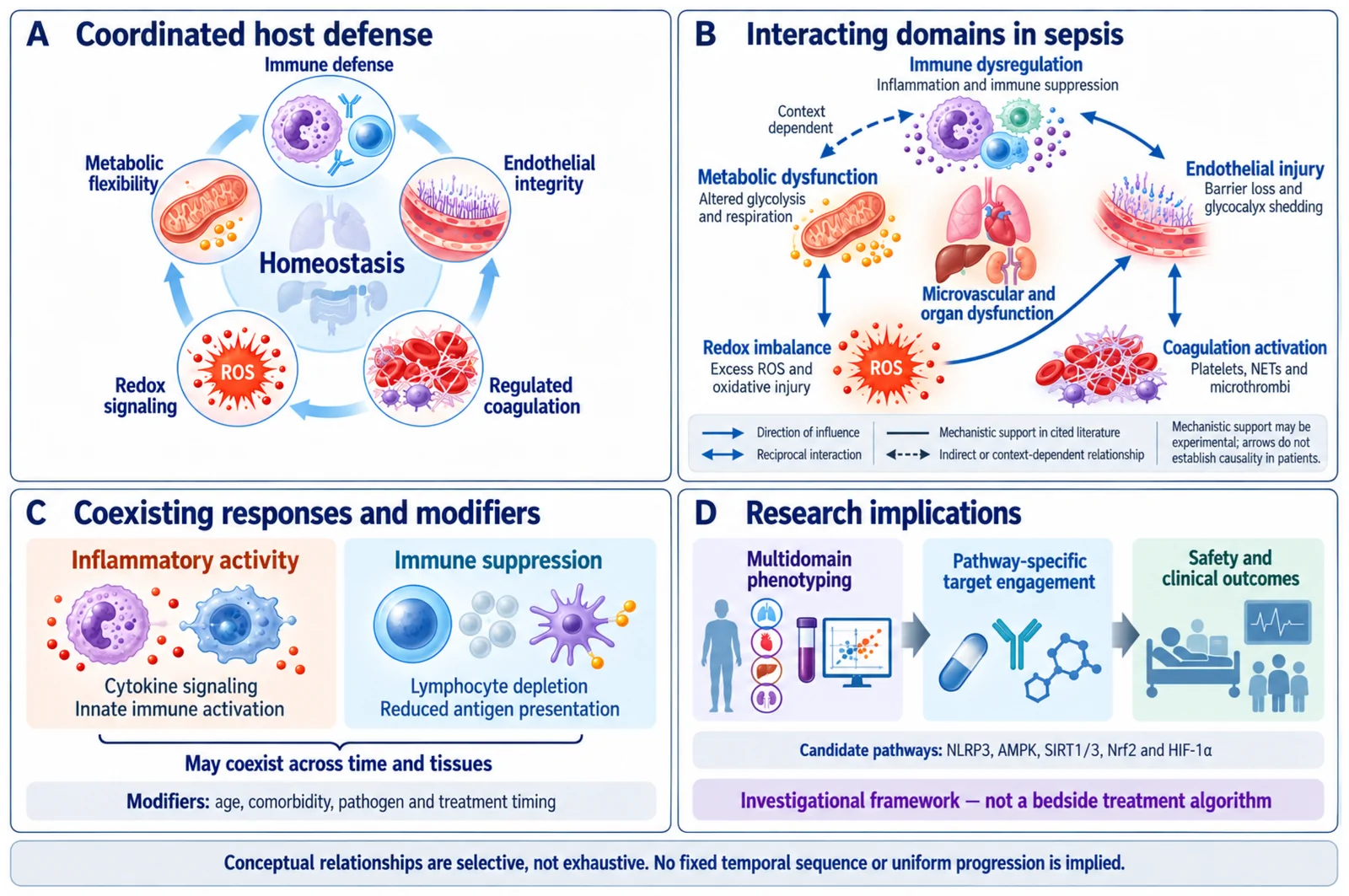
Integrated immune, endothelial, coagulation, redox, and metabolic dysregulation in sepsis. (**A**) Coordinated host-defense functions supporting homeostasis. (**B**) Selected interactions among five host-response domains associated with microvascular and organ dysfunction. Solid arrows indicate relationships with mechanistic support in the literature, which may derive from experimental studies; dashed arrows indicate indirect or context-dependent relationships. Single-headed and double-headed arrows represent directional and reciprocal influences, respectively, without establishing causality in patients or a fixed temporal sequence. (**C**) Inflammatory activity and immune suppression may coexist, with their expression influenced by age, comorbidity, pathogen characteristics, and treatment timing. (**D**) Research implications linking multidomain phenotyping to pathway-specific target engagement, safety assessment, and clinical outcomes. The framework is conceptual and does not constitute a validated bedside treatment algorithm. AMPK, AMP-activated protein kinase; HIF-1α, hypoxia-inducible factor-1α; NETs, neutrophil extracellular traps; NLRP3, NOD-like receptor family pyrin domain-containing 3; Nrf2, nuclear factor erythroid 2–related factor 2; ROS, reactive oxygen species; SIRT1/3, sirtuins 1 and 3. Created in BioRender. Américas, U. (2026) https://BioRender.com/9064736.

Sepsis can therefore be conceptualized as failure of the integrated host response, in which inflammatory, redox, metabolic, coagulation, and endothelial programs lose functional coordination [56,57]. Modulation of shared inflammatory and metabolic signaling pathways may eventually offer advantages over blockade of a single mediator, but its clinical value remains uncertain. [58,59]. Figure 1 summarizes the conceptual framework.

## 4. Endothelial Dysfunction as a Common Pathway of Organ Injury

### 4.1. Endothelial Dysfunction, Coagulation Activation, and Microvascular Thrombosis

The endothelium is a biologically active organ that regulates vascular permeability, vasomotor tone, leukocyte trafficking, platelet activity, coagulation, and fibrinolysis. In sepsis, endothelial cells transition from a quiescent antithrombotic phenotype toward an activated, permeable, proadhesive, and procoagulant state. This transition is characterized by glycocalyx degradation, altered nitric oxide signaling, disruption of endothelial junctions, mitochondrial dysfunction, and increased expression of adhesion molecules [17,18,22].

Complement activation contributes to this process through generation of bioactive fragments such as C3a and C5a, which promote leukocyte recruitment, endothelial activation, inflammatory signaling, and vascular permeability [19,22]. Neutrophil extracellular traps provide a scaffold for platelet adhesion and coagulation while exposing histones, proteases, and chromatin capable of directly injuring the endothelial surface [21]. Platelets likewise function as inflammatory effectors by interacting with activated leukocytes and endothelial cells and by releasing vasoactive, inflammatory, and prothrombotic mediators [21,22].

These processes reinforce one another and can promote microvascular thrombosis. Endothelial injury promotes leukocyte and platelet adhesion, coagulation activation, and capillary obstruction; the resulting regional hypoperfusion and cellular stress further reinforce inflammatory and endothelial activation [20,21,22,29].

Loss of glycocalyx integrity and dysregulation of the angiopoietin–Tie2 axis further reduce barrier stability [36,37,60]. Functionally, these abnormalities produce heterogeneous capillary perfusion, impaired oxygen diffusion, interstitial edema, and regional ischemia despite apparently restored systemic hemodynamics. This framework also helps explain the coexistence of thrombocytopenia or bleeding risk with microvascular thrombosis.

Endothelial injury should therefore be regarded as both a consequence and a propagator of septic pathophysiology. Nevertheless, sepsis should not be described exclusively as a vascular disease, because epithelial barriers, parenchymal cells, immune compartments, the nervous system, and organ-specific responses also contribute to organ failure. The endothelium therefore plays a central role in the interplay between inflammation, coagulation activation, microcirculatory dysfunction, and tissue injury.

### 4.2. Persistent Endothelial Dysfunction

Experimental evidence suggests that endothelial dysfunction may persist after partial resolution of the acute infectious and inflammatory response [61]. In cecal ligation and puncture models, vascular tissues obtained from sepsis-surviving animals have demonstrated impaired endothelium-dependent relaxation, enhanced contractile responses to angiotensin II, and reduced nitric oxide bioavailability [61]. These abnormalities have been associated with persistent ROS generation, reduced antioxidant activity, tyrosine nitration, and activation of the RhoA–ROCK pathway, which increases calcium sensitization in vascular smooth muscle [61,62].

Persistent vascular dysfunction may also involve endothelial senescence and sustained platelet, leukocyte, and coagulation activation. Experimental studies have reported activation of p53–p21-associated senescence programs and sustained expression of adhesion molecules such as ICAM-1 and VCAM-1 [62]. Platelet-derived extracellular vesicles may reinforce this phenotype by promoting neutrophil extracellular trap formation, endothelial activation, and procoagulant signaling.

Together, these findings suggest that endothelial injury may evolve into a self-maintaining state involving oxidative stress, altered vasoreactivity, senescence, and immunothrombosis. Such mechanisms could contribute to persistent microcirculatory abnormalities and delayed organ recovery after apparent control of infection. However, most evidence derives from experimental models, and the duration, prevalence, and clinical relevance of persistent endothelial dysfunction in human sepsis survivors remain incompletely defined.

These findings provide mechanistic support for longitudinal investigation of endothelial function during and after sepsis, but they do not establish a therapeutic indication in humans.

### 4.3. Emerging Experimental Pathways in Sepsis-Associated Endothelial Dysfunction

The pathways discussed in this section were included because experimental evidence links them directly to endothelial barrier or microvascular dysfunction in sepsis and because their biological activity could potentially be assessed using mechanistic biomarkers or pharmacodynamic readouts. Their inclusion does not imply equivalent translational maturity or current clinical applicability.

#### 4.3.1. Extracellular Vesicles and Exosome-Based Approaches

Extracellular vesicles, including exosomes, transport proteins, lipids, messenger RNAs, and microRNAs between cells [63]. In experimental sepsis, their effects on endothelial activation, oxidative stress, apoptosis, and barrier integrity depend on their cellular origin and molecular cargo [64,65]. Clinical translation is limited by variability in isolation methods and composition, as well as unresolved questions regarding biodistribution, dosing, manufacturing reproducibility, immunogenicity, and procoagulant risk [63].

#### 4.3.2. miR-126 and Endothelial Repair

miR-126 contributes to endothelial integrity, angiogenic signaling, inflammatory regulation, and cell survival. Experimental studies suggest that its modulation may reduce vascular permeability and support endothelial repair in sepsis [66,67].

However, circulating miR-126 concentrations may not accurately reflect intracellular activity, and targeted endothelial delivery without systemic or off-target effects has not been established [66,67,68].

#### 4.3.3. Autophagy Modulation

Autophagy contributes to cellular stress adaptation by removing damaged proteins and mitochondria and by regulating inflammatory signaling. Experimental modulation of autophagy can affect endothelial survival, mitochondrial turnover, inflammasome activation, and immune-cell function in sepsis [17,69].

Its effects are nevertheless dependent on dose, timing, organ involvement, and host phenotype, while agents such as rapamycin also influence broader mTOR-dependent immune and metabolic pathways [17,69,70].

#### 4.3.4. TRP Channels and Calcium-Dependent Endothelial Dysfunction

TRP channels regulate calcium entry in endothelial and immune cells. Experimental activation of TRPV4 and TRPM7 can induce endothelial contraction, disrupt VE-cadherin-associated junctions, and increase vascular permeability [71,72,73]. Calcium-dependent signaling may also influence inflammatory activation, neutrophil migration, and reactive oxygen species generation [71,74].

**Figure 2:**
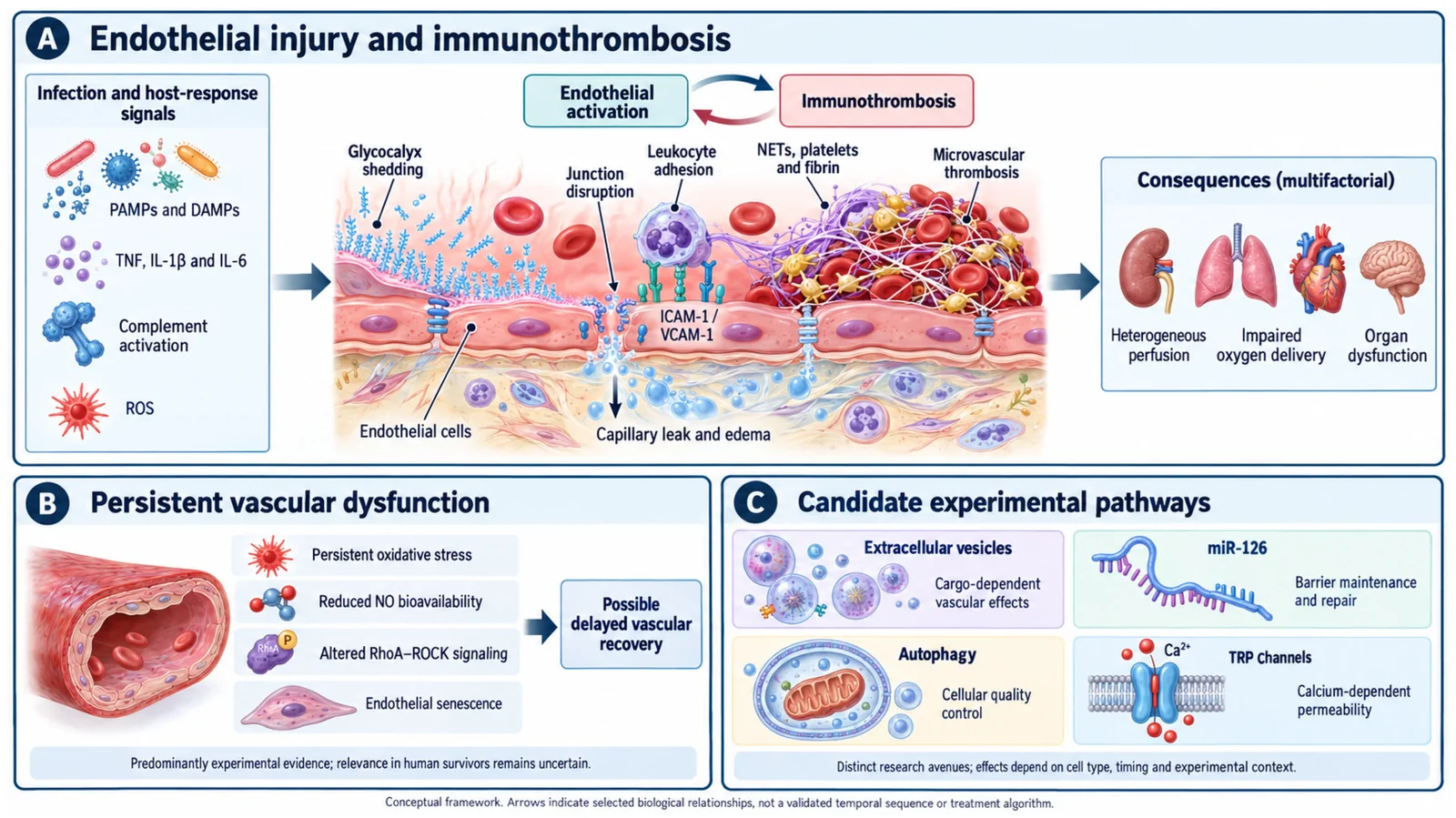
Endothelial dysfunction linking inflammation, immunothrombosis, and organ injury in sepsis. (**A**) Infection-associated and host-derived signals promote endothelial activation, glycocalyx shedding, junction disruption, and leukocyte adhesion. Reciprocal interactions between endothelial activation and immunothrombosis contribute to microvascular thrombosis, capillary leak, and heterogeneous perfusion. These processes contribute to organ dysfunction within a broader, multifactorial response. (**B**) Experimental evidence suggests that persistent oxidative stress, reduced nitric oxide bioavailability, altered RhoA–ROCK signaling, and endothelial senescence may delay vascular recovery; their relevance in human sepsis survivors remains uncertain. (**C**) Extracellular vesicles, miR-126, autophagy, and TRP channels represent distinct experimental research avenues, not a sequential pathway or established therapeutic strategy. Arrows illustrate selected biological relationships rather than a validated temporal sequence. DAMPs, damage-associated molecular patterns; ICAM-1, intercellular adhesion molecule-1; IL, interleukin; miR-126, microRNA-126; NETs, neutrophil extracellular traps; NO, nitric oxide; PAMPs, pathogen-associated molecular patterns; RhoA, Ras homolog family member A; ROCK, Rho-associated coiled-coil-containing protein kinase; ROS, reactive oxygen species; TNF, tumor necrosis factor; TRP, transient receptor potential; VCAM-1, vascular cell adhesion molecule-1. Created in BioRender. Américas, U. (2026) https://BioRender.com/awj597j.

Together, these effects link abnormal calcium signaling to endothelial barrier failure and inflammatory amplification. However, the broad distribution of TRP channels and their tissue- and phase-dependent functions make selective pharmacological targeting difficult. Modulation may produce unintended vascular, renal, pulmonary, or immune effects [71,75]. TRP channels should therefore remain mechanistic targets for investigation rather than candidates for routine therapeutic use. Figure 2 summarizes the endothelial and immunothrombotic mechanisms discussed in this section.

## 5. Biomarkers for Serial Host-Response Assessment

Biomarkers can serve different purposes. Prognostic markers estimate outcome risk; predictive markers identify a differential response to a specific treatment; and pharmacodynamic markers indicate whether an intervention affected its intended pathway. A prognostic association alone should not be interpreted as evidence for treatment selection, which requires prospective demonstration of a treatment-by-biomarker interaction [10].

Serial research assessment may integrate immune competence, inflammatory activity, endothelial and coagulation abnormalities, and metabolic status. These domains overlap but are not interchangeable. Their value lies in examining coexisting abnormalities and temporal changes and informing prospectively designed, mechanism-matched trials, rather than assigning each patient a fixed biological label [34].

### 5.1. Immune and Inflammatory Biomarkers

Monocyte HLA-DR expression and absolute lymphocyte count are the two immune selection criteria most frequently used in clinical studies of immunotherapy for sepsis-induced immunosuppression. However, studies vary markedly in their definitions, sampling times, thresholds, and outcomes; therefore, no universal value currently defines sepsis-induced immunosuppression or mandates immunostimulatory treatment [14].

Additional measurements, including ex vivo stimulated cytokine release, T-cell phenotyping, checkpoint expression, and serial circulating cytokines, may improve biological characterization. Nevertheless, they require standardized protocols and rapid turnaround, and none are currently validated as stand-alone predictors of response to routine immunomodulatory therapy. Their present role is mainly in research phenotyping and biomarker-enriched trial design [76].

### 5.2. Endothelial and Immunothrombotic Biomarkers

Syndecan-1 and soluble thrombomodulin reflect complementary aspects of endothelial injury. Syndecan-1 is commonly interpreted as a marker of glycocalyx shedding, whereas soluble thrombomodulin may indicate endothelial-cell injury and disruption of endogenous anticoagulant pathways [77]. Increased circulating concentrations have been associated with disease severity, organ dysfunction, microvascular injury, and adverse outcomes in sepsis [78,79].

These biomarkers may reveal vascular injury that is not captured by systemic blood pressure, cardiac output, or conventional resuscitation targets. Nevertheless, neither syndecan-1 nor soluble thrombomodulin has a universally accepted diagnostic threshold or validated treat-to-target range. Their concentrations may be influenced by renal function, comorbid endothelial disease, sampling time, assay characteristics, and non-septic causes of vascular injury [77,78,79]. They may contribute to risk stratification and research-based characterization of endothelial injury, but they are not validated for selecting or adjusting endothelial-directed treatment.

The angiopoietin–Tie2 axis may provide additional information regarding endothelial activation and barrier destabilization. Increased angiopoietin-2 relative to angiopoietin-1 or impaired Tie2 signaling has been associated with vascular permeability and adverse outcomes. However, assay availability and absence of validated intervention thresholds remain major barriers to clinical implementation [60].

Platelet count, D-dimer, fibrinogen, conventional coagulation tests, and viscoelastic assays may describe components of coagulation dysfunction and immunothrombosis. These measurements are clinically relevant but are not specific markers of endothelial injury or microvascular thrombosis. They should be interpreted in conjunction with infection severity, hepatic function, disseminated intravascular coagulation, bleeding risk, and platelet consumption. Similarly, mottling and bedside microcirculatory assessments may provide indirect evidence of perfusion abnormalities but cannot independently define the biological mechanism responsible for them.

### 5.3. Metabolic and Mitochondrial Biomarkers

Lactate is an established marker of circulatory and metabolic stress, but its biological interpretation is multidimensional. Elevated lactate may result from impaired oxygen delivery, adrenergically accelerated glycolysis, mitochondrial dysfunction, altered pyruvate metabolism, or reduced hepatic and renal clearance [13,80]. Serial lactate kinetics may therefore provide clinically useful information regarding overall physiological response, but lactate alone cannot distinguish mitochondrial failure from hemodynamic or clearance abnormalities.

Direct measurements of cellular bioenergetics, including oxygen-consumption rate and extracellular acidification rate, may characterize the relative contribution of oxidative metabolism and glycolytic flux [16]. The OCR/ECAR relationship can provide mechanistic information regarding metabolic flexibility, mitochondrial reserve, and cellular stress. However, values are highly dependent on cell type, isolation procedures, assay platforms, substrate conditions, and timing. A low or high OCR/ECAR ratio should not be interpreted uniformly across monocytes, neutrophils, lymphocytes, endothelial cells, or platelets.

Mitochondrial membrane potential, mitochondrial ROS production, ATP generation, mitochondrial DNA release, and respiratory-chain activity represent additional candidate readouts. These measures may identify specific components of mitochondrial injury, but most require research-grade platforms and lack standardized reference ranges. Furthermore, mitochondrial adaptation may be protective in one cellular compartment and harmful in another, limiting the validity of a single systemic “mitochondrial dysfunction” category.

Metabolomic and transcriptomic approaches may improve identification of bioenergetic and redox phenotypes by integrating multiple pathways simultaneously. Their principal limitations include cost, analytical complexity, delayed results, platform dependence, and uncertain reproducibility across institutions. At present, their use is most appropriate for discovery studies and prospective validation rather than immediate bedside decision-making.

### 5.4. Composite Phenotyping and Serial Reassessment

No individual biomarker captures the complete host-response state. A biologically coherent phenotype would ideally combine functional immune measurements, circulating inflammatory trajectories, endothelial injury markers, metabolic readouts, and clinical context. Serial assessment is particularly important because a phenotype observed at admission may change after antimicrobial treatment, source control, resuscitation, organ support, or progression of the disease.

Composite phenotyping should not be reduced to a binary classification. More useful categories for research may include:

- A predominantly inflammatory–endothelial activation profile;
- A predominantly immunosuppressed profile;
- A predominantly bioenergetic or metabolic dysfunction profile;
- A mixed profile involving abnormalities across several domains;
- An indeterminate profile in which available measurements are insufficient for biological classification.

These profiles are hypothesis-generating research constructs that require prospective definition and validation; they should not be interpreted as validated diagnostic phenotypes, bedside classification tools, or treatment-selection algorithms. Their purpose is not to replace clinical assessment or established sepsis management, but to enrich clinical trials and test whether particular biological patterns predict response to specific host-directed interventions [34,76]. For research purposes, phenotype assignment should rely on prospectively prespecified biomarker criteria and sampling windows rather than on qualitative clinical judgment alone. Individual biomarker domains should be classified as positive, negative, or unavailable according to trial-specific criteria derived, where possible, from previously published studies. Participants meeting predefined criteria for more than one biological profile should be classified as having a mixed profile rather than being forced into a single category. An indeterminate profile should be assigned when required measurements are unavailable, technically uninterpretable, or insufficient to support a predefined biological classification. These proposed rules are intended for prospective testing in biomarker-enriched trials and should not be interpreted as validated diagnostic criteria for clinical practice [34,76].

Where previous sepsis trials have used biomarker-based enrichment, published thresholds should be considered study-specific examples rather than universal diagnostic cutoffs. For instance, mHLA-DR < 8000 antibodies bound per cell has been used to identify sepsis-associated immunosuppression in GM-CSF trials, whereas the IRIS-7 trial used an absolute lymphocyte count ≤ 900 cells/µL to define severe lymphopenia [81,82]. Baseline biomarker sampling should be obtained before administration of the investigational intervention and within a prospectively prespecified enrollment window; for the proposed framework, we suggest obtaining baseline measurements within 24 h before randomization or first study-drug administration. Subsequent sampling intervals should be predefined according to the pharmacokinetic and pharmacodynamic characteristics of the intervention. Real-world implementation requires prioritization according to assay availability, turnaround time, technical complexity, and relevance to the intervention under study. Routinely available measures such as absolute lymphocyte and platelet counts, lactate, conventional coagulation tests, and organ-function variables are more suitable for a feasible core assessment. In contrast, serial mHLA-DR measurement may require standardized flow-cytometric processing and rapid laboratory access, while mitochondrial bioenergetic assays and advanced molecular profiling remain largely research-grade and may be more appropriate for predefined mechanistic substudies or centralized batch analysis. Autonomic measurements should likewise be interpreted cautiously because of their sensitivity to arrhythmias, ventilation, sedation, vasoactive therapy, and disease severity. Accordingly, repeated advanced testing should be limited to measurements that are directly linked to the trial hypothesis or expected target engagement rather than performed routinely in all participants. Candidate biomarker domains and their principal limitations are summarized in Table 1.

Note that the assignment of individual biomarkers to screening/enrichment or mechanistic/pharmacodynamic roles should be prospectively defined according to the intervention, biological target, assay characteristics, and trial objective. The same biomarker should not be assumed to serve both purposes without protocol-specific justification. Patient-centered outcomes constitute a distinct third level and should not be replaced by evidence of biomarker or target-engagement improvement. The biomarkers and quantitative values presented in Table 1 are illustrative research examples and should not be interpreted as validated diagnostic or treatment-selection thresholds. Where numerical cutoffs are provided, they represent study-specific trial criteria rather than universal clinical standards. For most candidate biomarkers, validated predictive cutoffs, treatment-selection AUCs, and effect-size estimates remain unavailable or insufficiently standardized across studies.

## 6. Host-Directed Strategies: Evidence and Translational Maturity

The interventions discussed in this section differ in their mechanisms, supporting evidence, and stage of clinical development. They include approaches that modify neural regulation, inflammatory resolution, inflammasome activation, mitochondrial function, oxidative stress responses, or immune function. Biological plausibility and changes in laboratory markers do not by themselves demonstrate clinical benefit. Except where specifically stated, these interventions remain investigational [76,83].

Host-directed interventions must not delay or replace established sepsis management, including timely antimicrobial therapy, source control, hemodynamic stabilization, and organ support [84]. Their evaluation in clinical trials requires prospectively defined eligibility criteria, appropriate timing, evidence that the intervention affected its intended biological pathway, preservation of antimicrobial defense, and improvement in clinically meaningful patient outcomes.

### 6.1. Neuroimmunomodulation Through the Vagal–α7nAChR Axis

The inflammatory reflex links the nervous and immune systems. Afferent pathways convey peripheral inflammatory signals to central autonomic networks, whereas efferent vagal pathways regulate immune activity through splenic and peripheral neural circuits [85,86,87,88]. The α7 nicotinic acetylcholine receptor is expressed by macrophages, dendritic cells, and selected lymphocyte populations and has been implicated in regulation of NF-κB, AP-1, and JAK/STAT-associated signaling [12,83,85,88].

In experimental models, activation of this pathway can attenuate TNF, IL-1β, and IL-6 production and influence leukocyte trafficking, endothelial activation, and autonomic–immune communication. These findings support the investigation of electrical vagal stimulation and pharmacological α7nAChR modulation [85]. However, whether these effects can be reproduced in human sepsis without compromising pathogen clearance remains uncertain.

Evidence remains largely preclinical and translational. Clinical application is complicated by autonomic instability, sedation, vasoactive therapy, mechanical ventilation, cardiac arrhythmias, and variability in stimulation protocols. Heart-rate variability may provide an exploratory autonomic readout, but it is not a validated surrogate for vagal target engagement or treatment response. Neuroimmunomodulation should therefore be evaluated in controlled translational studies with predefined safety, infection-control, and clinical endpoints.

### 6.2. Specialized Pro-Resolving Mediators

Specialized pro-resolving mediators include resolvins, protectins, and maresins derived from polyunsaturated fatty acids. They promote active resolution by limiting excessive neutrophil recruitment, enhancing efferocytosis, regulating macrophage function, supporting endothelial repair, and facilitating tissue recovery [89,90].

Unlike broad anti-inflammatory suppression, active resolution is intended to terminate inflammation while preserving antimicrobial defense. Experimental studies suggest that SPMs influence NF-κB signaling, oxidative injury, apoptosis, macrophage function, and endothelial integrity. Their effects, however, vary with the mediator, receptor, dose, route of administration, tissue compartment, and stage of disease [90,91].

Translation to clinical use is limited by incomplete pharmacokinetic and pharmacodynamic characterization, rapid endogenous metabolism, formulation challenges, receptor-specific effects, and the lack of validated markers of target engagement. Changes in cytokines or efferocytosis-related measurements may indicate biological activity, but they do not establish improvements in organ recovery, infection control, or survival. SPM-based treatment therefore remains an early translational strategy rather than an established therapy [89,90].

### 6.3. Selective Modulation of the NLRP3 Inflammasome

The NLRP3 inflammasome integrates microbial and cellular danger signals and promotes caspase-1 activation, maturation of IL-1β and IL-18, gasdermin-mediated pore formation, and pyroptotic cell death [11,53,54,92]. Excessive activation can amplify inflammation, disrupt endothelial integrity, increase vascular permeability, and contribute to organ dysfunction.

Selective NLRP3 inhibition could limit inflammasome-mediated tissue injury while avoiding some of the broader effects of nonspecific cytokine suppression. However, inflammasome activity also contributes to antimicrobial defense, and the consequences of inhibition are likely to vary with the pathogen, infection source, cellular compartment, and timing of treatment. Excessive or mistimed blockade could impair microbial clearance or disrupt compensatory immune pathways.

Current evidence comes mainly from experimental models and early translational studies. Reductions in IL-1β, IL-18, caspase-1 activity, or pyroptosis-related markers may indicate target engagement, but no validated NLRP3-defined phenotype, treatment threshold, or therapeutic window has been established. NLRP3 modulation should therefore be tested in biomarker-enriched trials with rigorous monitoring of infection control and immune safety.

### 6.4. Mitochondrial Support and Restoration of Metabolic Flexibility

Mitochondrial-directed strategies seek to preserve respiratory capacity, remove damaged mitochondria, support biogenesis, and restore regulated ROS signaling. Potential targets include AMPK, SIRT1, SIRT3, Nrf2, mitophagy pathways, mitochondrial dynamics, and antioxidant-response systems [13,54,93,94].

Activation of AMPK and sirtuin-associated pathways may support mitochondrial turnover, respiratory function, substrate adaptation, endothelial resilience, and antioxidant responses, whereas Nrf2 activation may strengthen endogenous cytoprotective programs without indiscriminately suppressing ROS. Yet these pathways are pleiotropic and context-dependent: activation may be beneficial in one cell type or disease phase while impairing immune effector function in another.

The therapeutic goal is to restore metabolic flexibility rather than force all immune cells toward oxidative phosphorylation. Glycolysis supports rapid antimicrobial activity, whereas oxidative metabolism may favor cellular persistence, repair, and resolution. The optimal response therefore depends on cell type, functional demand, and disease phase. Similarly, HIF-1α may promote inflammatory glycolytic reprogramming while also supporting adaptation to hypoxia and cellular survival.

Potential pharmacodynamic measures include OCR, ECAR, mitochondrial reserves, membrane potential, ATP production, and oxidative-stress markers. Most remain research-grade measurements and are insufficiently standardized for treatment selection, while evidence for mitochondrial-support agents is still predominantly preclinical. Future studies should confirm cell-specific target engagement and determine whether metabolic improvement translates into organ recovery and patient-centered benefit.

### 6.5. Immunorestorative Therapies for Sepsis-Associated Immunosuppression

IL-7, granulocyte–macrophage colony-stimulating factor, and immune-checkpoint modulation are being investigated in patients with persistent immune dysfunction. Unlike anti-inflammatory therapies, these approaches aim to restore lymphocyte, monocyte, or antigen-presenting-cell function.

IL-7 promotes lymphocyte survival, proliferation, and functional recovery. Early studies have reported increases in circulating lymphocyte counts and other evidence of biological activity, but consistent improvements in mortality, organ recovery, or secondary infection rates have not been demonstrated [95,96]. An increase in lymphocyte number should therefore be interpreted as a pharmacodynamic response rather than evidence of clinical efficacy.

GM-CSF may enhance monocyte function and antigen presentation, particularly in patients selected according to reduced monocyte HLA-DR expression or impaired innate immune responsiveness. Studies have demonstrated restoration of selected immune biomarkers, but effects on patient-centered outcomes remain uncertain [45,97]. The optimal dose, duration, enrichment threshold, and safety-monitoring strategy have yet to be defined.

Blockade of inhibitory pathways such as PD-1/PD-L1 may reverse elements of T-cell exhaustion or anergy. Preclinical and clinical observational evidence supports continued investigation of this approach in carefully selected patients, but the risk of excessive immune activation, inflammatory injury, autoimmunity, or disruption of infection control remains important [48,49,98,99]. Evidence derived from oncology should not be extrapolated directly to sepsis, where the duration, intensity, and biological context of checkpoint activation differ substantially.

These therapies should remain confined to controlled trials using prospectively defined criteria for sepsis-associated immunosuppression. Enrichment may incorporate persistent lymphopenia, reduced monocyte HLA-DR, impaired ex vivo cytokine responsiveness, checkpoint upregulation, or recurrent secondary infection. Because each marker has limited specificity, patient selection should rely on a combination of findings rather than a single criterion.

### 6.6. Glucocorticoids and Nonspecific Antioxidant Approaches

Intravenous glucocorticoids are an established adjunctive treatment for selected patients with ongoing vasopressor-dependent septic shock. Their use is based on persistent shock rather than on routine cortisol testing or a validated immune phenotype [84].

Glucocorticoids influence inflammatory gene transcription, vascular responsiveness, glucose metabolism, and neuroendocrine signaling [100,101,102,103]. Because these effects are broad and are not specific to a defined sepsis phenotype, glucocorticoid treatment should not be described as restoration of a coordinated host response or as biomarker-guided immunotherapy. Treatment requires monitoring for adverse effects, particularly hyperglycemia and neuromuscular weakness.

Future redox-directed therapy should aim to limit irreversible oxidative injury while preserving physiological ROS signaling. Activation of endogenous pathways such as Nrf2 may offer greater biological coherence than high-dose nonspecific antioxidant administration, but efficacy and safety remain insufficiently established in human sepsis.

### 6.7. Safety, Timing, and Therapeutic Windows

The effect of any host-directed intervention is likely to depend on timing, biological context, dose, and duration. Nevertheless, fixed universal windows such as 0–6 h, 6–24 h, or greater than 24 h should not be presented as validated treatment phases. Patients may enter sepsis at different points in their biological trajectory, and inflammatory, immunosuppressive, endothelial, and metabolic abnormalities may coexist.

Therapeutic windows should therefore be defined prospectively within each study according to:

- Time from recognition and source-control intervention;
- Baseline host-response phenotype;
- Severity and trajectory of organ dysfunction;
- Persistence of infection;
- Pharmacokinetic and pharmacodynamic characteristics;
- Serial evidence of target engagement;
- Predefined safety and withdrawal criteria.

Biological response should not be inferred from a single marker. For example, an increase in monocyte HLA-DR may suggest restoration of antigen presentation but does not ensure improved pathogen clearance or survival. Similarly, reduced IL-6 may reflect target engagement without demonstrating clinical benefit. Candidate biomarkers should be treated as pharmacodynamic readouts until their ability to guide initiation, escalation, or withdrawal is prospectively validated.

The principal candidate interventions and their translational maturity are summarized in Table 2.

## 7. A Biomarker-Enriched Translational Framework for Precision Pharmacology

The main translational challenge is not the lack of candidate immunomodulatory agents, but identifying which patients are most likely to benefit, when treatment should begin, how target engagement should be assessed, and when therapy should be modified or withdrawn. Precision pharmacology in sepsis therefore requires the prospective integration of dynamic phenotyping with pharmacokinetic, pharmacodynamic, and clinical outcome data.

### 7.1. Principles of Dynamic Phenotyping

Established sepsis care must remain the immediate priority, including prompt antimicrobial therapy, source control, hemodynamic stabilization, and organ support. Research phenotyping may proceed in parallel, provided that it does not delay these interventions [52,84].

At baseline, patients enrolled in biomarker-enriched studies could undergo assessment across several complementary biological domains:

- Inflammatory activity;
- Innate and adaptive immune competence;
- Endothelial injury;
- Coagulation and immunothrombosis;
- Circulatory and metabolic stress;
- Cellular bioenergetics.

The purpose is not to assign patients a fixed biological label, but to identify the dominant and potentially modifiable abnormalities present at a given time. Because inflammatory, immunosuppressive, endothelial, and metabolic disturbances may coexist, a patient may meet criteria for more than one candidate profile. An indeterminate category should also be retained when the available data do not permit confident classification [33,34,104,105].

Candidate inflammatory–endothelial activation profiles could be characterized by persistent inflammatory mediator elevation, endothelial injury markers, capillary leak, platelet or coagulation activation, and microcirculatory abnormalities [17,18,60]. Candidate immunosuppressed profiles could include persistent lymphopenia, reduced monocyte HLA-DR expression, diminished ex vivo cytokine responsiveness, impaired antigen presentation, viral reactivation, or increased inhibitory checkpoint expression [14,45,48,49,76,98]. Candidate metabolic or bioenergetic profiles could involve abnormal cellular respiration, reduced mitochondrial reserve, membrane-potential abnormalities, or persistent metabolic stress that is not fully explained by macrocirculatory dysfunction [16,105].

These proposed profiles are hypothesis-generating constructs that require prospective definition and validation. They should not be inferred from a single routine laboratory value, interpreted as mutually exclusive disease states, or used for bedside treatment selection. Their purpose is to generate and prospectively test biological enrichment hypotheses in controlled studies evaluating pathway-specific interventions [33,34,40,52,104,105].

### 7.2. Treat-to-Target as a Research Paradigm

Treat-to-target designs may be useful in clinical trials, although the proposed biological targets are not yet validated for routine decision-making. Within a controlled study, the sequence could include:

- Initiation of established sepsis care;
- Baseline multidomain host-response phenotyping;
- Enrollment according to prospectively defined biological criteria;
- Administration of a trial-specific intervention;
- Serial measurement of pharmacological exposure, biological target engagement, and safety;
- Reassessment of the host-response profile;
- Continuation, modification, or withdrawal according to predefined protocol rules;
- Evaluation of patient-centered clinical outcomes.

Potential pharmacodynamic responses could include recovery of monocyte HLA-DR expression, improved ex vivo cytokine responsiveness, increased lymphocyte number or function, favorable endothelial biomarker trajectories, or improvement in cellular respiration measurements [33,34,95,96,97,104,105]. These biological changes should be evaluated together with pathogen clearance, infection recurrence, organ function, adverse events, and survival. Improvement in an isolated biomarker cannot establish therapeutic success if the intervention impairs microbial control, exacerbates organ dysfunction, or produces clinically relevant toxicity [48,49,95,96,97].

Biomarkers used for trial enrichment should also be distinguished from pharmacodynamic readouts. An enrichment biomarker identifies patients considered more likely to exhibit the targeted biological abnormality, whereas a pharmacodynamic biomarker assesses whether the intervention affected its intended pathway. Neither category should automatically be interpreted as a validated surrogate for clinical benefit [34,95,96,104,105].

Combination or escalation strategies should not be presumed superior to monotherapy. Although broader mechanistic coverage may be attractive, combining interventions can increase toxicity, pharmacological interactions, and interpretive complexity. Sequential or combination approaches should therefore be tested only after each component has demonstrated adequate exposure, target engagement, and acceptable safety [26,27,34,104,105].

Sequential host-directed strategies introduce important attribution challenges because changes observed after a second intervention may reflect the effect of the first intervention, the second intervention, their interaction, or the natural evolution of the host response. We therefore propose that sequential trials use prospectively defined transition criteria, repeat phenotyping before each treatment change, and measure intervention-specific pharmacodynamic markers before and after each therapeutic step. When combination therapy is evaluated, designs capable of estimating the contribution of individual components, such as factorial or appropriately controlled multi-arm designs, should be preferred when feasible [34,106]. Combination therapy should not proceed solely because multiple biological abnormalities coexist; each component should have a clear mechanistic rationale and prior evidence of acceptable exposure, target engagement, and safety.

Safety monitoring should also be intervention-specific and prospectively defined. At minimum, host-directed trials should assess serious adverse events, new or worsening organ dysfunction, pathogen clearance, persistence or recurrence of infection, secondary infections, and relevant immune-function changes [45,95]. For immunostimulatory strategies, monitoring should include evidence of excessive inflammatory activation or worsening organ injury, whereas interventions that suppress inflammatory pathways require particular attention to impaired microbial control and secondary infection. Prespecified criteria for treatment interruption, withdrawal, or escalation should be incorporated into the protocol, and biomarker improvement alone should not justify continuation of therapy in the presence of clinical deterioration or relevant toxicity [95,96].

### 7.3. Proposed Conceptual Framework

**Figure 3:**
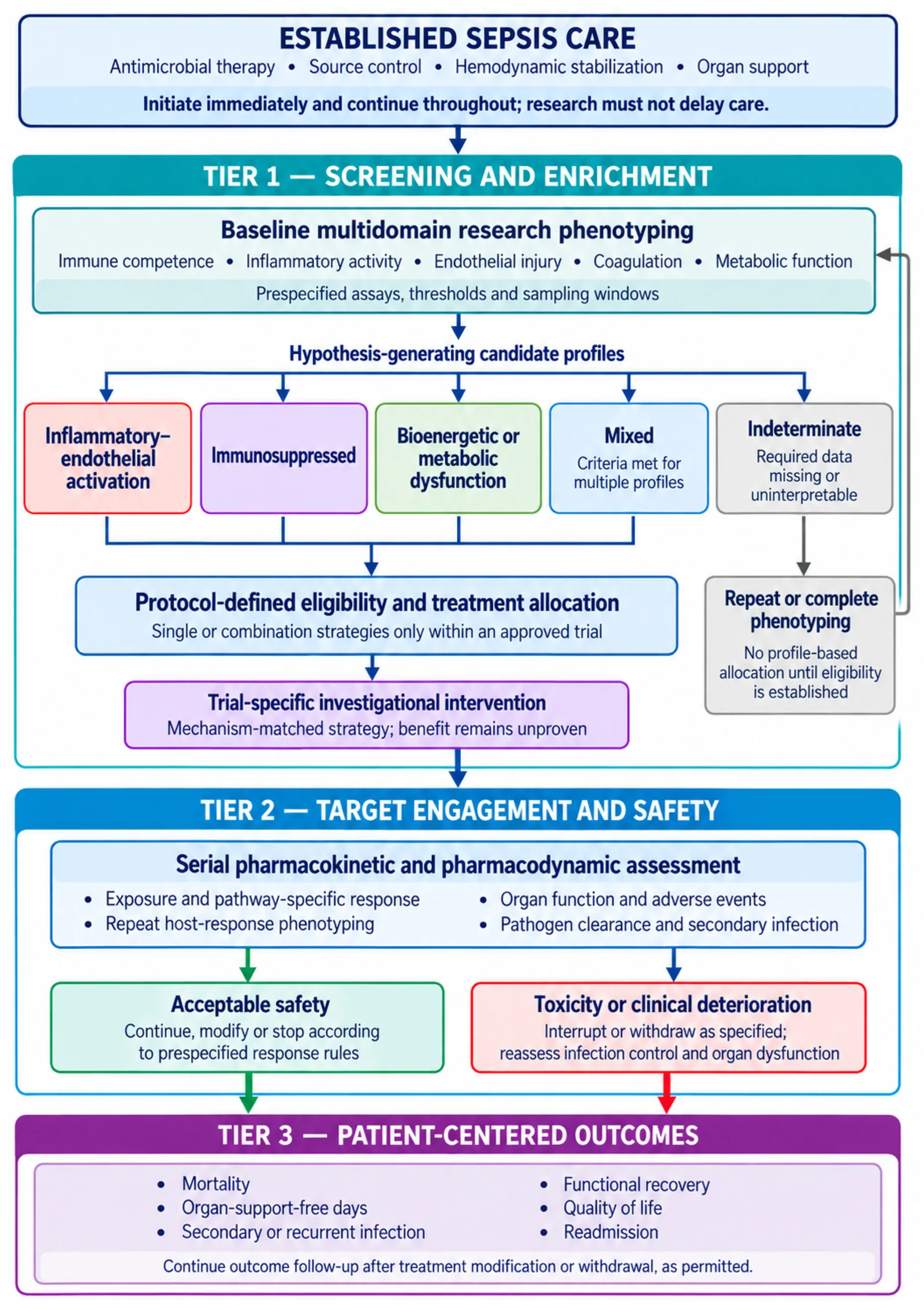
Tiered biomarker-enriched trial framework for host-directed interventions in sepsis. Established sepsis care continues independently of research procedures. Tier 1 comprises multidomain screening, candidate profile identification, and protocol-defined eligibility and allocation. Candidate profiles include inflammatory–endothelial activation, immunosuppressed, bioenergetic or metabolic dysfunction, mixed, and indeterminate profiles. Indeterminate profiles require additional assessment before profile-based allocation. Tier 2 comprises serial pharmacokinetic, pharmacodynamic, target-engagement, and safety assessments, with treatment decisions governed by prespecified rules. Tier 3 evaluates patient-centered outcomes, including follow-up after treatment modification or withdrawal where permitted. These profiles remain hypothesis-generating research constructs. Biomarker improvement or target engagement should not be interpreted as a surrogate for clinical benefit unless prospectively validated. Created in BioRender. Américas, U. (2026) https://BioRender.com/ey7ps3i.

Figure 3 presents a biomarker-enriched research framework rather than a bedside treatment algorithm. Established antimicrobial, source-control, hemodynamic, and organ-supportive care remains the first priority. Multidomain phenotyping is subsequently used to define candidate biological profiles for prospective trial enrollment and serial pharmacodynamic reassessment [33,34,40,52,84,104].

## 8. Discussion

The principal contribution of this review is a trial-design framework that connects multidomain host-response assessment with biological enrichment, mechanism-specific target engagement, safety monitoring, and patient-centered outcomes. Its purpose is to make these relationships explicit and testable, rather than to establish a new clinical classification. A central requirement is to distinguish biomarkers associated with prognosis from those that identify differential treatment effects; the latter require the prospective evaluation of treatment-by-biomarker interactions [33,34,104,105].

Clinical translation depends on the maturity of both the intervention and the selection strategy. Early randomized trials of IL-7 have demonstrated biological activity, including lymphocyte recovery [82,95], but such findings do not establish consistent patient-centered benefit. Other candidate approaches remain at heterogeneous stages of preclinical, translational, or early clinical development. Adjunctive glucocorticoids for persistent vasopressor-dependent septic shock remain distinct because their established use is not based on the proposed research profiles [84]. Consequently, the framework does not justify routine phenotype-directed treatment or empirical combinations.

Several limitations constrain interpretation. This structured narrative review did not include formal risk-of-bias assessment or certainty grading and remains susceptible to selective inclusion and interpretation. Its evidence base combines clinical studies with animal and in vitro investigations, including indirect evidence from other conditions. Assay methods, sampling windows, enrichment thresholds, and phenotype definitions vary across studies [14,34,76]. The proposed profiles and their transitions therefore remain hypotheses requiring prospective validation; conceptual connections between domains should not be interpreted as a demonstrated causal sequence in patients.

Future studies should prioritize a limited number of biologically justified measurements and prespecified decisions. Enrichment criteria, sampling schedules, intervention-specific pharmacodynamic endpoints, and safety rules should be defined before recruitment. Sequential and combination trials additionally require designs that can distinguish component effects and interactions from spontaneous recovery or changes in standard care. We propose that clinical evaluation prioritize mortality, organ support, infection-related outcomes, functional recovery, and quality of life, with continued outcome follow-up after treatment discontinuation where permitted. We also recommend prespecifying analyses of treatment-effect heterogeneity according to baseline comorbidity and immune status, rather than relying on unplanned subgroup comparisons [34,84,95,104,105].

Operational feasibility is equally important. Table 3 distinguishes a routinely available core assessment from trial-specific assays and advanced mechanistic substudies. It provides illustrative sampling and reporting targets, rules for missing measurements, assay-harmonization requirements, and a staged approach to incorporating complex technologies. These implementation levels describe operational complexity and are distinct from the functional tiers in Table 1 and Figure 3. Their purpose is to support testable protocols while ensuring that research procedures do not delay established sepsis care.

## 9. Conclusions

Sepsis involves interacting immune, endothelial, coagulation, redox, and metabolic abnormalities. The endothelium is a major interface linking complement, NETs, platelets, glycocalyx injury, microvascular thrombosis, and organ dysfunction.

Serial multidomain measurements can test whether these abnormalities coexist or recover at different rates, but they are not validated treatment targets. Neuroimmunomodulation, pro-resolving mediators, NLRP3 inhibition, mitochondrial-directed strategies, IL-7, GM-CSF, and checkpoint inhibition remain investigational or at an early phase. Their clinical value depends on prospectively defined eligibility, confirmed target engagement, infection-related safety, and improvement in patient-centered outcomes.

Biomarker-guided host-response modulation should therefore be regarded as a clinical-trial strategy rather than current bedside care.

## Figures and Tables

**Table 1:** Tiered research toolkit for biomarker-guided host-response assessment in sepsis.

Biological Domain/Assessment Level	Candidate Readouts	Potential Biological Interpretation	Main Limitations	Proposed Research Use	Primary Role in the Tiered Research Toolkit
Innate immune competence	Monocyte HLA-DR (published trial precedent: <8000 antibodies bound per cell for two consecutive days) [81]; ex vivo LPS-stimulated cytokine production	Antigen-presentation capacity, monocyte responsiveness, and endotoxin tolerance	Assay variability; no universal thresholds; specialized processing; limited rapid availability	Biological enrichment and serial pharmacodynamic monitoring	Tier 1—Screening/enrichment when prospectively defined for eligibility; Tier 2—Target engagement when measured serially after intervention
Adaptive immune competence	Absolute lymphocyte count (published trial precedent: ≤900 cells/µL in IRIS-7) [82]; T-cell subsets; proliferation or functional assays; PD-1/PD-L1 and other checkpoint expression	Lymphocyte depletion, impaired adaptive function, or functional exhaustion	Lymphopenia is nonspecific; complex immunophenotyping; tissue–blood discordance	Identification of candidate immunosuppressed profiles	Tier 1—Screening/enrichment for candidate immunosuppressed phenotypes; Tier 2—Target engagement for serial immune-response assessment
Inflammatory activity	IL-6, TNF, IL-1β, IL-10, and procalcitonin trajectories	Magnitude and temporal evolution of inflammatory and infection-associated signaling	Timing dependence; limited mechanistic specificity; influence of organ clearance and previous treatment	Serial assessment with other biomarker domains and risk stratification	Primarily Tier 2—Mechanistic/pharmacodynamic assessment; use for Tier 1 enrichment requires prospectively defined trial-specific criteria
Endothelial injury and barrier dysfunction	Syndecan-1; soluble thrombomodulin; angiopoietin-2/angiopoietin-1 or Tie2-related measurements	Glycocalyx shedding, endothelial-cell injury, anticoagulant-pathway disruption, and barrier destabilization	Assay heterogeneity; renal and comorbidity effects; no validated intervention thresholds	Endothelial-profile enrichment and mechanistic monitoring	Tier 1—Screening/enrichment for predefined endothelial profiles; Tier 2—Target engagement through serial endothelial trajectories
Immunothrombosis and coagulation	Platelet count; D-dimer; fibrinogen; conventional coagulation tests; viscoelastic assays; selected microcirculatory indices	Coagulation activation, consumption, fibrinolytic abnormalities, and possible microvascular dysfunction	Low mechanistic specificity; affected by liver disease, bleeding, disseminated intravascular coagulation, and treatment	Assessment of coagulation status and treatment safety	Tier 1—Baseline characterization/safety assessment and Tier 2—Serial pathway and safety monitoring
Circulatory and metabolic stress	Lactate concentration and kinetics	Integrated circulatory, adrenergic, metabolic, and clearance-related stress	Cannot distinguish tissue hypoxia, mitochondrial dysfunction, adrenergic glycolysis, or impaired clearance	Standard clinical monitoring and trial stratification	Tier 1—Baseline clinical characterization/stratification and Tier 2—Serial physiological response assessment
Cellular bioenergetics	OCR, ECAR, OCR/ECAR relationship, mitochondrial reserve, membrane potential, ATP production, mitochondrial ROS, and circulating mitochondrial DNA	Respiratory capacity, glycolytic activity, metabolic flexibility, and mitochondrial stress	Research-grade platforms; cell-type dependence; preanalytical variability; no standardized reference ranges	Mechanistic studies and candidate pharmacodynamic assessment	Primarily Tier 2—Mechanistic target-engagement assessment; currently suited mainly to research-grade or advanced substudy platforms
Integrated molecular profiling	Transcriptomics, proteomics, metabolomics, and multidomain composite scores	Identification of molecular endotypes and interacting inflammatory, endothelial, and metabolic pathways	Cost; delayed turnaround; analytical complexity; platform dependence; need for external validation	Discovery, endotype definition, and biomarker-enriched trial design	Advanced Tier 1 research enrichment for endotype discovery/validation and Tier 2 mechanistic assessment; not currently suitable for routine real-time trial allocation
Autonomic regulation	Heart-rate variability and related autonomic measurements	Exploratory evidence of altered autonomic–immune regulation	Strongly affected by arrhythmias, ventilation, sedation, vasoactive drugs, and disease severity	Exploratory neuroimmune phenotyping	Primarily Tier 2—Exploratory mechanistic assessment; not established as a validated screening tool
Patient-centered outcome evaluation	Mortality; organ-support-free days; secondary or recurrent infection; functional recovery; quality of life; readmission	Clinical benefit, safety, recovery, and longer-term patient impact	Influenced by baseline severity, comorbidity, competing risks, and duration of follow-up; biomarker improvement does not necessarily predict clinical benefit	Evaluation of treatment efficacy and clinical relevance	Tier 3—Patient-centered outcomes. These outcomes should remain distinct from screening/enrichment and target-engagement biomarkers

**Table 2:** Candidate host-directed strategies under investigation in sepsis.

Candidate Strategy	Principal Biological Node	Candidate Phenotype or Pharmacodynamic Readouts	Translational Maturity	Major Limitations and Safety Considerations	Host-Defense Monitoring Endpoint
Vagal or α7nAChR modulation	Neural–immune regulation; NF-κB/AP-1-associated signaling; autonomic tone	Inflammatory–endothelial activation profile; cytokine trajectories; exploratory autonomic measurements	Predominantly preclinical and translational evidence	Uncertain target engagement; effects of sedation, ventilation, vasoactive drugs, and arrhythmias; infection-control safety	Microbiological clearance and secondary infection, when assessed in clinical trials
Specialized pro-resolving mediators	Active inflammatory resolution, efferocytosis, endothelial repair, and macrophage reprogramming	Resolution-related mediators; efferocytosis markers; inflammatory and endothelial trajectories	Preclinical to early translational evidence	Incomplete PK/PD; rapid metabolism; formulation and receptor-specific effects; absence of established clinical efficacy	Microbiological clearance and secondary infection, particularly when immune-resolution pathways are modified
Selective NLRP3 inhibition	Caspase-1 activation; IL-1β and IL-18 maturation; pyroptosis	Inflammasome-activation markers; IL-1β, IL-18, caspase-1, and pyroptosis-related readouts	Predominantly preclinical evidence	Potential impairment of pathogen defense; pathogen-, compartment-, and phase-dependent effects; no validated selection threshold	Pathogen clearance and secondary infection
AMPK–SIRT1/SIRT3–Nrf2 modulation	Mitochondrial turnover and respiratory function, endogenous antioxidant response, and metabolic adaptation	Bioenergetic or metabolic dysfunction profile; OCR/ECAR, respiratory reserve, membrane potential, and oxidative-stress markers	Predominantly preclinical and mechanistic evidence	Pleiotropic and cell-specific effects; research-grade assays; uncertain dose and therapeutic window	No intervention-specific host-defense endpoint established; infection-related safety should remain part of trial monitoring
Mitophagy and mitochondrial-support strategies	Removal of damaged mitochondria, mitochondrial biogenesis, and restoration of respiratory function	ATP production, mitochondrial ROS, membrane potential, mitochondrial DNA, and respiratory-chain activity	Preclinical to early translational evidence	Heterogeneous interventions; uncertain cell-specific effects; no validated systemic mitochondrial phenotype	No intervention-specific host-defense endpoint established; infection-related safety should remain part of trial monitoring
IL-7	Lymphocyte survival, proliferation, and functional recovery	Persistent lymphopenia; impaired T-cell function; lymphocyte recovery	Early-phase human evidence with pharmacodynamic activity	Clinical outcome benefit unproven; risk of excessive activation; patient-selection criteria not standardized	Pathogen clearance; secondary or recurrent infection; lymphocyte recovery and T-cell function as immune-function endpoints
GM-CSF	Monocyte and antigen-presenting-cell function	Reduced monocyte HLA-DR; impaired ex vivo innate immune responsiveness	Early clinical and translational evidence	Heterogeneous trial results; uncertain effect on mortality or infection; dose and duration unresolved	Pathogen clearance; secondary infection; monocyte HLA-DR recovery and ex vivo innate immune responsiveness as immune-function endpoints
PD-1/PD-L1 modulation	Reversal of inhibitory checkpoint signaling	Checkpoint upregulation; impaired T-cell function; persistent immunosuppressed profile	Early-phase safety and pharmacodynamic evidence	Inflammatory or autoimmune toxicity; infection-related safety; clinical efficacy unproven	Pathogen clearance; secondary or recurrent infection; T-cell functional recovery and checkpoint-related immune recovery
Targeted redox modulation	Endogenous redox regulation and protection from irreversible oxidative injury	Oxidative-stress trajectories; mitochondrial and phagocyte-function readouts	Predominantly preclinical evidence	Risk of impairing physiological ROS signaling and antimicrobial oxidative burst; no validated target range	Pathogen clearance and phagocyte antimicrobial function, where feasible
Intravenous glucocorticoids	Vascular responsiveness and broad transcriptional modulation	Persistent vasopressor-dependent septic shock; hemodynamic response and adverse-event monitoring	Established adjunctive clinical use in selected patients	Not a biomarker-selected host-directed therapy; hyperglycemia, neuromuscular, metabolic, and infection-related adverse effects	Secondary infection and microbiological control as safety outcomes

Note: Except for intravenous glucocorticoids used as adjunctive therapy in selected patients with vasopressor-dependent septic shock, the strategies listed remain investigational. Biomarker changes are candidate enrichment or pharmacodynamic measures and should not be considered validated surrogate clinical outcomes. α7nAChR, α7 nicotinic acetylcholine receptor; ECAR, extracellular acidification rate; GM-CSF, granulocyte–macrophage colony-stimulating factor; OCR, oxygen-consumption rate; PK/PD, pharmacokinetics/pharmacodynamics; SPMs, specialized pro-resolving mediators.

**Table 3:** Pragmatic implementation model for biomarker-enriched host-directed trials in sepsis.

Implementation Level	Trial Component	Core Requirements	Timing and Decision Rules
A. Core assessment	Routine clinical and laboratory panel	Lymphocyte and platelet counts, lactate, coagulation tests, organ function, and documentation of infection management and organ support.	Baseline within 24 h before randomization and before treatment; repeat at 24 h, 72 h, and day 7 or earlier discharge.
B. Trial-specific assays	Biological enrichment	Mechanism-related biomarkers with prespecified assays, thresholds, and repeat-confirmation criteria.	Results available before allocation; illustrative turnaround ≤ 24 h, subject to enrollment and confirmation requirements.
B. Trial-specific assays	Extended phenotyping and target engagement	Selected immune, endothelial, coagulation, or functional measures linked to the intervention.	Baseline and prespecified pharmacodynamic time points. Decision-guiding results must precede treatment changes; other assays may be batched.
C. Advanced studies	Mechanistic substudies	Selected cellular respiration, mitochondrial, molecular, or autonomic assays addressing predefined hypotheses.	Protocol-specific sampling with validated processing and storage. Delayed results do not guide real-time allocation.
Across A–C	Missing measurements	Record reasons, distinguish death from discharge, and prespecify statistical handling. Missingness does not indicate recovery.	Missing eligibility data: indeterminate profile pending reassessment. Missing required safety data: protocol-defined hold or reassessment.
Across A–C	Assay harmonization	Shared procedures, calibration, reporting units, sample-acceptance criteria, and interlaboratory quality control.	Site qualification before recruitment; ongoing checks of assay performance and processing/reporting times.
Across A–C	Staged assay introduction	Begin with core and essential eligibility assays; add complex tests as substudies before evaluating decision-guiding use.	Prespecified stages or approved amendments. No retrospective redefinition of allocation using delayed exploratory results.

**Note:** Timing targets are illustrative design proposals, not validated standards. Follow-up times are measured from the first investigational dose and should be adapted to intervention-specific pharmacology, assay requirements, and safety needs. Implementation levels A–C describe operational complexity, whereas Tiers 1–3 in Table 1 and Figure 3 distinguish screening/enrichment, target engagement, and clinical outcomes. Research procedures must not delay established sepsis care.

## Data Availability

No new data were created or analyzed in this study. Data sharing is not applicable to this article.

## Abbreviations

The following abbreviations are used in this manuscript:

ROS	reactive oxygen species
TNF	tumor necrosis factor
IL-1β	interleukin-1 beta
IL-6	interleukin-6
NLRP3	NOD-like receptor family pyrin domain containing 3
AMPK	AMP-activated protein kinase
SIRT	sirtuin
Nrf2	nuclear factor erythroid 2-related factor 2
HIF-1α	hypoxia-inducible factor 1 alpha
α7nAChR	alpha-7 nicotinic acetylcholine receptor
SPMs	specialized pro-resolving mediators
NF-κB	nuclear factor kappa B
AP-1	activator protein 1
JAK/STAT	Janus kinase/signal transducer and activator of transcription
DHA	docosahexaenoic acid
EPA	eicosapentaenoic acid
IL-18	interleukin-18
ATP	adenosine triphosphate
ΔΨm	mitochondrial membrane potential
HPA	hypothalamic–pituitary–adrenal (axis)
GR	glucocorticoid receptor
PCT	procalcitonin
LPS	lipopolysaccharide
OCR	oxygen consumption rate
ECAR	extracellular acidification rate
HRV	heart rate variability
Rv	resolvins
PD	protectins
MaR	maresins
CTLA-4	cytotoxic T-lymphocyte–associated protein 4
PK/PD	pharmacokinetics/pharmacodynamics
RCTs	randomized controlled trials
SOFA	Sequential Organ Failure Assessment
ICU	intensive care unit
QOL	quality of life
ROTEM	rotational thromboelastometry
TEG	thromboelastography
